# Skeletal nutrient vascular adaptation induced by external oscillatory intramedullary fluid pressure intervention

**DOI:** 10.1186/1749-799X-5-18

**Published:** 2010-03-11

**Authors:** Hoyan Lam, Peter Brink, Yi-Xian Qin

**Affiliations:** 1 Department of Biomedical Engineering, Stony Brook University, Bioengineering Building Stony Brook, NY 11794, USA; 2 Department of Physiology and Biophysics, Stony Brook University Basic Sciences, Building Stony Brook, NY 11794, USA

## Abstract

**Background:**

Interstitial fluid flow induced by loading has demonstrated to be an important mediator for regulating bone mass and morphology. It is shown that the fluid movement generated by the intramedullary pressure (ImP) provides a source for pressure gradient in bone. Such dynamic ImP may alter the blood flow within nutrient vessel adjacent to bone and directly connected to the marrow cavity, further initiating nutrient vessel adaptation. It is hypothesized that oscillatory ImP can mediate the blood flow in the skeletal nutrient vessels and trigger vasculature remodeling. The objective of this study was then to evaluate the vasculature remodeling induced by dynamic ImP stimulation as a function of ImP frequency.

**Methods:**

Using an avian model, dynamics physiological fluid ImP (70 mmHg, peak-peak) was applied in the marrow cavity of the left ulna at either 3 Hz or 30 Hz, 10 minutes/day, 5 days/week for 3 or 4 weeks. The histomorphometric measurements of the principal nutrient arteries were done to quantify the arterial wall area, lumen area, wall thickness, and smooth muscle cell layer numbers for comparison.

**Results:**

The preliminary results indicated that the acute cyclic ImP stimuli can significantly enlarge the nutrient arterial wall area up to 50%, wall thickness up to 20%, and smooth muscle cell layer numbers up to 37%. In addition, 3-week of acute stimulation was sufficient to alter the arterial structural properties, i.e., increase of arterial wall area, whereas 4-week of loading showed only minimal changes regardless of the loading frequency.

**Conclusions:**

These data indicate a potential mechanism in the interrelationship between vasculature adaptation and applied ImP alteration. Acute ImP could possibly initiate the remodeling in the bone nutrient vasculature, which may ultimately alter blood supply to bone.

## Introduction

Bone mass and morphology accommodates changes in mechanical demands by regulating the site-specific remodeling processes which consist of resorption of bone, typically followed by bone formation. The active processes of bone remodeling are responsible for bone turnover, repair, and regeneration [1,2]. Yet, the uncoupling of bone formation and resorption can have serious consequences, as demonstrated by stress fractures in military recruits and athletes [3-6]. The mechanical influence on bone adaptation remains a key issue in determining the etiology of stress injury to bone. From mechanotransduction point of view, bone remodeling is regulated by various parameters within the mechanical milieu, i.e., strain magnitude, frequency, duration, rate, and cycle number [7,8] Stress injuries were initially thought to emerge from repetitive vigorous activity, inducing an accumulation of fatigue microfractures and resulting in material failure [9]. However, this hypothesis of repetitive loading related fatigue microdamage as the sole causative factors for stress injuries has been shown to be inconsistent based on two key findings: a) the number of loading cycles associated with stress fracture in recruits and athletes are well below the fatigue fracture threshold, and that there is not enough duration for an accumulation of microdamage to contribute to material failure within the early onset of stress fractures [9-11], and b) the stress fracture site tends to occur close to the neutral axis of bending at the mid-diaphysis rather than the site with maximum strain magnitude [12,13].

The skeletal vascular system supplies nutrients to and remove wastes from bone tissue, marrow cavity and periosteum, in which blood flow is directly coupled with general status of bone health. The vasculature also regulates intramedullary pressure (ImP) via circulation. The principal nutrient artery pierces the diaphysis at the nutrient foramen, penetrates through the cortex and branch proximally and distally within the medullary cavity to the metaphyseal regions, supplying the inner two-thirds of the cortex [14,15]. During fracture healing, the amount of bone remodeling was significantly reduced if intracortical fluid flow, along with blood flow, was prevented [12,13]. There exist a close correlation between systemic blood pressure and ImP under the normal conditions. In various animal models, the ImP is approximately ranged 20-30 mmHg, while nearby systemic blood pressure is about 100-140 mmHg, which is approximately 4 folds higher than associated ImP (Table 1). Under the external loading condition, ImP is increased and/or alternated [13,16-20]. In a rat hindlimb disuse model, increased ImP by 68% via femoral vein ligation could significant increase the femoral bone mineral content and trabecular density [21]. Others have shown that increasing pressure gradient within the vasculature can induce new bone formation at the periosteal, endosteal and trabecular surfaces [14]. External skeletal muscle contraction can substantially increase ImP and subsequently enhance bone adaptation, even in a disuse model [18,22-24]. Mechanical intervention through vibratory knee joint loading can trigger bone formation. These experiments have evidently verified the critical role of the change in fluid pressure within the marrow cavity and the skeletal vasculature on bone adaptation [14,25] (Table 2).

Skeletal vasculature remodeling is critical for maintaining adequate tissue perfusion and is responsible for regulating interstitial fluid pressure. Arterial adaptation is often associated with hypertrophy of the vessel, redistribution of the extracellular matrix and smooth muscle cells (SMCs) [26,27]. The tunica media is the thickest layer in nutrient artery, which comprises of layers of SMCs embedded in a network of connective tissue. This layer provides tensile strength, elasticity and contractility to the vessel [28]. Its structure and morphology also play a critical role in maintaining blood pressure [28,29]. In human hypertension, histological analyses showed that there is a greater media/lumen ratio in untreated hypertensive subjects [30]. The greater media/lumen ratio is a result of either higher vessel wall area and/or smaller lumen area, or both.

**Table 1:** Blood pressure and nearby ImP.

*Animal*	*Blood Pressure (mmHg)*			*ImP (mmHg)*
Dog [23,33,50]	110-140	Femoral, Carotid arteries	17-40	Femoral diaphysis and metaphysic (mean)
Rabbit [23]	~120	Carotid artery	20-20	Femoral diaphysis (mean)
Rat [16,18]	20-30	Femoral arteries	5	Femoral marrow (peak-peak)
Turkey [19]	40-80	Ulna and femoral arteries	15-25	Ulna and femoral marrow (peak-peak)

**Table 2:** ImP induced by mechanical stimulation

*Animal*	*Location*	*Type of loading*	*ImP (mmHg) (peak-peak)*
Turkey [7,13,19]	ulna	~600 με axial	90-150
Rat [20]	femur	Venous ligation	60
Rat [16,18]	femur	Muscle stimulation	40
Rat [2]	femur	Knee loading	22

It is hypothesized that bone fluid flow induced by ImP can regulate the nutrient arterial adaptation. Thus, the objective of this study is to evaluate nutrient vessel remodeling under dynamic stimulation by evaluating the morphologic changes on the nutrient arterial wall with increased mechanical-induced ImP, and to discuss their potential role in regulating fluid flow through nutrient vessels,

## Methods

### Animals and Experimental Preparations

All surgical and experimental protocols were approved by the University's Lab Animal Use Committee. The surgical protocol was previously described and modified slightly for this study [13,19]. In brief, under isoflurane anesthesia, surgical procedures were performed on both left and right ulnae of twenty-nine adult skeletally mature male turkeys. For the left ulna, a 3-mm diameter hole was drilled and tapped through the cortex of the dorsal side, approximately 2 cm from the proximal end. A specially designed fluid loading device, with an internal fluid chamber approximately 0.6 cm^3^, was inserted into the bone with an O-ring seal to prevent leakage. The fluid loading device was attached to a surgical plastic tube with an inner diameter approximately 2 mm wide and 12 cm in length, filled with saline as external oscillatory loading fluid. A diaphragm was placed in the center of the fluid chamber, separating the internal marrow from external oscillatory loading fluid. The bone marrow and external flow media were completely isolated from one another to avoid contamination and infection. The plastic tube extended through the skin and coupled the fluid loading device to the oscillatory loading unit. The external portion of the device was flushed and cleaned each day, while antibiotic cream was applied to the surrounding tissue to further prevent infection. The contra-lateral right ulnae served as sham control. With similar surgical procedures as the left, a 3-mm diameter hole was drilled and tapped at the proximal end of the right cortex. A titanium screw with an O-ring was used in replacement of the fluid loading device.

Additional four turkeys were sacrificed at the end of the experiment without undergo any surgical operation. These age-matched controls were needed to examine the handedness, if any, between the left and right ulnae.

### Dynamic Fluid Flow Stimulation

The loading system was calibrated based on previous study 13. In brief, with the same surgical procedure as above, an additional tube was connected at the distal end of the ulnae, where a 50-psi pressure transducer (Entran EPX-101W) was placed into the marrow cavity. The ImP was measured within the physiological magnitude of 10-180 mmHg and at a range of frequency, 1-40 Hz. A standard graph of marrow pressure at different frequencies was generated and was used to calibrate the loading system.

After surgery, the animals were monitored closely during normal activities. Fluid pressure stimulation began on the second day subsequent to the surgery. A sinusoidal fluid pressure was applied to the marrow cavity of the left ulna through an external fluid oscillatory loading unit. The loading unit was controlled to generate changes in the fluid pressure within the intramedullary canal, by varying magnitudes and frequencies. Based on the calibration data, the pressure magnitudes applied were between 50 mmHg to 90 mmHg, which have shown to be under physiological range [7,13,19]. The sinusoidal ImP was applied for 10 minutes per day, 5 days per week, at 3 Hz for 3-weeks (n = 7), 3 Hz for 4-weeks (n = 5), 30 Hz for 3-weeks (n = 6), and 30 Hz for 4-weeks (n = 11).

### Histomorphometry Analyses

**Figure 1:**
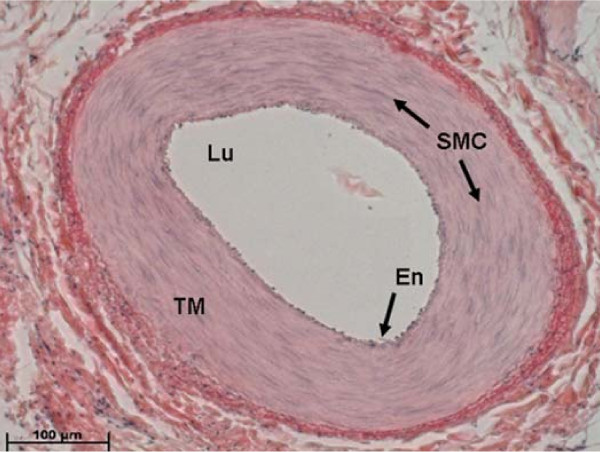
**A representative cross-sectional image of a nutrient artery from turkey ulna**. TM, tunica media; L, lumen; E, endothelial cells; SMC, smooth muscle cells. The scale bar at the bottom left corner is 100 μm.

Immediately after the animals were sacrificed, the principle nutrient arteries from both left and right ulnae were located. Under the microscope, the nutrient arteries were carefully dissected starting from the lumen with approximately 8 mm in length, and fixed in 10% formalin solution immediately. The adaptive responses of the arteries were analyzed through a standard soft tissue histology procedure. The fixed arteries were embedded in paraffin wax. In order to obtain arterial cross sections, each vessel was oriented so that it was straight and perpendicular to the cutting surface. The paraffin blocks were then sectioned transversely to produce 8 μm thin slices (RM2165 Microtome, Leica, IL). Each section was stained with hematoxin and eosin (H&E, Polyscience, PA), dehydrated with a series of ethanol and cleared with xylene. A representative H&E stained cross sectional nutrient artery image is shown in Figure 1.

The arterial wall area, lumen area, and wall thickness of each section were measured using OsteoMeasure (Version 2.2, SciMeasure, GA) by manually contouring the inner and outer boundaries of the tunica media layer of the nutrient artery. Six cross-sections were analyzed for each nutrient artery. The full cross section of the nutrient artery was viewed under a digitized microscope at 40× magnification.

The SMC layer numbers were assessed via sector analysis, in which the arterial cross-section was divided into six equal sectors. An image was captured at each sector using an inverted microscope (Zeiss, AxioVision 4, Germany) under 200× magnification. The numbers of SMCs were quantified by drawing a line across the arterial wall, perpendicular to SMC stretch, and counting the nucleus along the line. All the analyses were performed by a single operator to ensure consistency of measurements.

### Statistical Analyses

Statistical Analysis System (SAS, Cary, NC) software was used for data analyses. Experimental data is expressed as means ± standard error (SE) of each group. The nonparametric Wilcoxon test was performed and significance level was considered at p < 0.05. Data from each histomorphometric parameter was compared in two ways: (1) each stimulated group was compared to the average of all controls (age-matched controls and sham) to show the effect of ImP on the nutrient arterial morphology, and (2) the ImP stimulated groups were compared between the various loading regimes to demonstrate the importance of the loading parameters.

## Results

### Nutrient Arterial Wall Area

**Figure 2:**
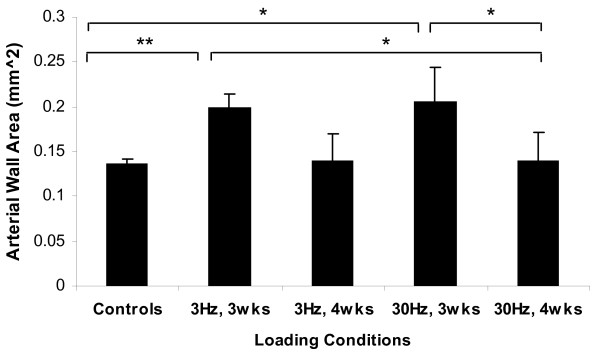
**Arterial wall area histomorphological analysis of the nutrient arteries, subjected to 3 Hz or 30 Hz ImP stimulation, 10 minutes/day, 5 days/week for 3-week or 4-week**. Comparison between the experimental arteries and the pooled average of age-matched and sham controls. Values are mean ± SE. Significant difference between the ImP stimulated nutrient artery and pooled controls (* p < 0.05 & ** p < 0.01).

The tunica media of the nutrient arteries demonstrated up to a 50% increase in area when subjected to ImP stimulation (Figure 2). It is important to point out that nutrient arteries from the age-matched animals showed an average of 4% natural differences in arterial wall area between the left and the right ulnae (0.121 ± 0.01 mm^2 ^vs. 0.126 ± 0.015 mm^2^), demonstrating minimal left and right handedness in turkeys. There was no significant difference between the age-matched controls and sham controls. Thus, the average of the age-matched and sham arterial wall area was calculated to serve as an overall control and compared that to each experimental wall area. The cross sectional arterial wall area for loading conditions at 3 Hz and 30 Hz for 3-weeks were 0.199 ± 0.2 mm^2 ^and 0.207 ± 0.3 mm^2^, which were significantly increased by 46% (p < 0.01) and 51% (p < 0.05), respectively, comparing to the control (0.136 ± 0.05 mm^2^). Furthermore, comparisons between experimental groups showed significant changes between ImP loadings applied at 30 Hz for 3-weeks versus 4-weeks and 3 Hz, 3-weeks vs. 30 Hz, 4-weeks (p < 0.05) (Figure 2). There was no significant change between experimental and control in 4-weeks loading for both 3 Hz and 30 Hz.

### Nutrient Arterial Lumen Area

**Figure 3:**
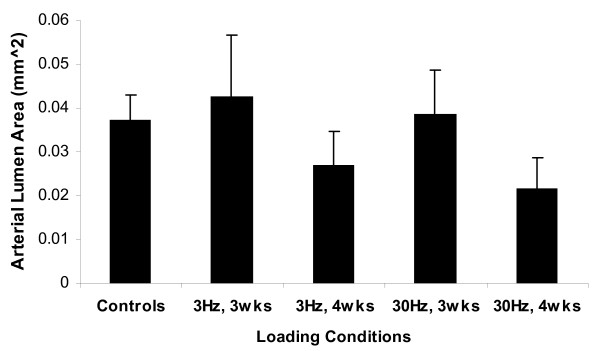
**Arterial lumen area histomorphological analysis of the nutrient arteries subjected to 3 Hz or 30 Hz ImP stimulation, 10 minutes/day, 5 days/week for 3-week or 4-week**. Comparison between the experimental arteries and the pooled average of age-matched and sham controls. Values are mean ± SE.

Similar to the arterial wall area, the difference in lumen area between the left and right ulnae in the age-matched animals was approximately 4%. The age-matched and sham data were pooled and compared to experimental groups. Fluid loadings for 3-weeks showed 14% and 3% increase in cross sectional lumen area at 3 Hz and 30 Hz stimulations when compared to controls, yielding lumen area of 0.045 ± 0.01 mm^2 ^and 0.039 ± 0.01 mm^2^, respectively (Figure 3). Yet, loadings for 4-weeks showed decrease in lumen area at both frequencies, yielding area of 0.027 ± 0.007 mm^2 ^for 3 Hz (-28%) and 0.022 ± 0.007 mm^2 ^for 30 Hz (-42%). Though the trends in lumen area changes were seen between 3-weeks and 4-weeks stimulations, no statistical significance was found within and between groups due to the large variability within the samples.

### Nutrient Arterial Wall Thickness

**Figure 4:**
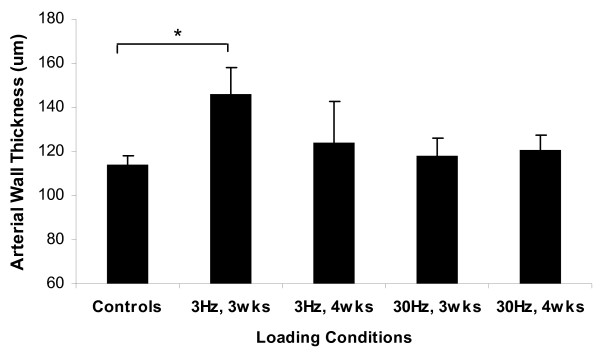
**Arterial wall thickness histomorphological analysis of the nutrient arteries subjected to 3 Hz or 30 Hz ImP stimulation, 10 minutes/day, 5 days/week for 3-week or 4-week**. Comparison between the experimental arteries and the pooled average of age-matched and sham controls. Mean ± SE (* p < 0.05).

The thickness of the tunica media increased for all loading conditions, ranging from 4-28% (Figure 4). The arterial wall thickness after 3-weeks stimulation at 3 Hz was 146 ± 11 μm significantly increased by 28% from the control, 113 ± 4 μm (p < 0.05). Although the percentage changes in thickness was not as great as those of area, it suggested that augment in thickness might partially contribute to the arterial wall area changes. Despite there was no significant difference between the sham and age-matched controls, the sham thickness value for the animals subjected to 3-weeks ImP loading at 30 Hz (100 ± 7 μm) was smaller than other controls (116 ± 7 μm) (P > 0.5). Thus, when compared to its sham operated arteries, the 30 Hz stimulation induced an 18% augmentation at the wall thickness. Lastly, ImP stimulation at 3 Hz and 30 Hz for 4-weeks showed a slight increase (9% and 6%, respectively) (P > 0.5), which may be due to the reduction of the lumen area seen in Figure 3.

### SMC Layer Numbers

**Figure 5:**
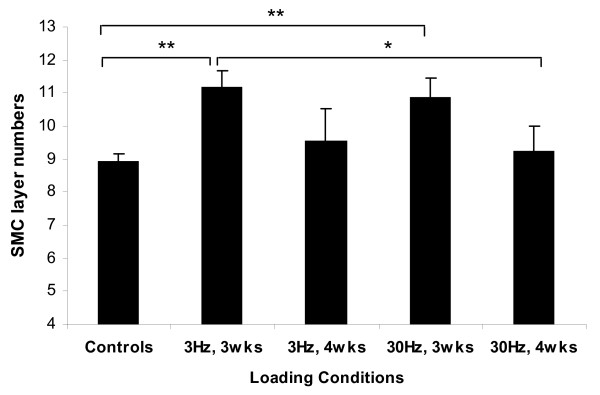
**SMC layer numbers analysis of the nutrient arteries subjected to 3 Hz or 30 Hz ImP stimulation, 10 minutes/day, 5 days/week for 3-week or 4-week**. Comparison between the experimental arteries and the pooled average of age-matched and sham controls. Mean ± SE (* p < 0.05 & ** p < 0.01).

The SMC layer numbers for each nutrient artery were quantified. The ImP stimulation at 3 Hz and at 30 Hz for 3-weeks showed a significant 25% and 22% increase, respectively, in SMC layer numbers when compare to the controls (25% and 22%, respectively, p < 0.01) (Figure 5). As mentioned before, arterial wall area and thickness were also augmented after ImP loading for 3-weeks. It is highly possible that such increase in SMC layer numbers was responsible for the alteration in arterial morphometry. No significance was observed for the 4-weeks stimulation. Further, comparisons between experimental groups showed significant changes between fluid loadings applied at 3 Hz for 3-weeks versus 30 Hz for 4-weeks (p < 0.05) (Figure 5).

## Discussion

The objective of this study was to examine how intramedullary pressure influenced nutrient artery remodeling. Previous experiments have implied that bone fluid flow is a mediator involved in bone remodeling by influencing bone cell activities through improper nutrient transport [7,13,19]. However, the mechanism in which bone fluid flow can lead to changes in nutrient transport is not clearly characterized. In this study, the morphological analyses of the nutrient arterial wall demonstrated that bone fluid flow induced by daily cyclic ImP stimulations has the potential to initiate nutrient artery remodeling, which may ultimately alter the blood supply to bone and ultimately affect the bone remodeling processes.

Previous in vivo studies have demonstrated repetitive mechanical loading generated ImP can alter interstitial fluid flow and initiate bone remodeling [7,13,19,31]. It is hypothesized that these ImP seriously impact bone fluid flow by collapsing the nutrient artery at the peak of each loading cycle, decreasing normal blood flow into the marrow cavity. Augmentation of femoral marrow pressure and interstitial fluid flow, induced by femoral vein ligation, could significantly influence bone quantity under functional disuse condition [20]. On the contrary, blockage of arterial supply led to the reduction of the nutrients and oxygen to bone [32,33]. The changes in oxygen and carbon dioxide levels in bone callus and bone necrosis, strengthening the idea that arterial occlusion can deplete nutrient supplies required for bone cells activities [34,35]. However, the maximal ImP value response to the impact loading in our previous avian model was 150 mmHg (~20 KPa). Compared to the load-generated solid phase matrix stress and strain, e.g., 10-100 MPa by 1,000 με, and the estimated fluid pressure at the value of 3 MPa [36], the applied direct ImP (50-90 mmHg) is in the physiological range. Such a small value of ImP will not collapse the vessels.

Mechanical forces related to the velocity of arterial blood flow have been shown to be important determinants of arterial structural changes [26,37,38]. Several experiments on the ligation of rat mesenteric bed had shown a 90% reduction of blood flow in the upstream arteries. This decrease in blood flow resulted in a 21% and 37% reduction in lumen diameter and vessel wall area [39,40]. Conversely, arteries exposed to over 100% in blood flow showed a marked elevation in lumen diameter (38%) and arterial wall area (58%) [39,40]. Thus, the 50% enlargement of the nutrient arterial wall area observed in this study (Figure 2) may be the result of the increase blood flow via the ImP stimulation.

Axial loading, e.g. generating peak 600 με, can amplify ImP 10-fold above the arterial pressure, e.g., from 18 mmHg to 150 mmHg, driving blood flow through the nutrient artery [12,13]. In a functional disuse model, bone loss was observed via the thinning of the cortex due to endosteal resorption and an increase in intracortical porosity. However, when external oscillatory fluid flow was applied to the marrow cavity, bone mass was significantly improved at the mid-diaphysis due to both periosteal and endosteal new bone formation [13]. This data clearly illustrated the effects of anabolic fluid flow in bone adaptation, which was capable of maintaining bone mass and likely to inhibit bone loss due to disuse.

In this study, the fluid magnitudes (50 mmHg to 90 mmHg) for the cyclic hydraulic stimulation applied to the marrow cavity were imposed at physiological levels. The frequencies were chosen to mimic the number of loading cycles relevant to physiological level and to a military training regimen, i.e., 3 Hz for 10 minutes provide 1,800 loading cycles and 30 Hz for 10 minutes provides 18,000 cycles. The ImP due to circulation in the turkey is approximately 18 mmHg and previous experiments have shown that ImP of 76 mmHg was sufficient to generate bone remodeling at 30 Hz [7,13]. The loading rate sensitivity of bone remodeling was also shown in recent disuse model under dynamic muscle contraction [16,18]. The duration of the experiments was also chosen based on previous studies stating that the risk of stress fractures occur at the early onset of training, with the rate of occurrence generally elevated by the third week of training [41].

Fluid loadings at 3 Hz and at 30 Hz for 3-weeks have generated the greatest changes in nutrient arterial wall area. This strongly implied that the duration of loading plays an important role in vessel remodeling; it is clear that 3-weeks of cyclic ImP stimulation was sufficient to initiate vessel wall remodeling with increase wall area, lumen area, wall thickness, and SMC layer number. Four-weeks of cyclic ImP stimulation are enough to trigger bone adaptation [13,16,18]. Together with the observations obtained from this study, where 3-weeks fluid loading resulted in the most morphological changes in the nutrient arteries, the results implied that the nutrient arteries adapt to the altered ImP precede and/or occur concurrently with the bone remodeling process. Hence, there is a strong implication that the adaptation of the nutrient arteries may serve as a critical mediator between bone fluid flow and bone remodeling.

Acute vasculature adaptation is impaired by endothelial pressure hypercholesterolemia (such as flow-mediated dilatation) and fluid wall shear stress. Previous works indicated that increased vascular flow results in adaptive vessel remodeling as dependant on applied shear stress [29,42]. Morphological changes occur rapidly following flow alteration and do not require chronic insult to affect substantial and significant structural transformation [29]. The results from this study indicated that 3 weeks ImP can significantly change the nutrient artery morphology, but such effects were attenuated in the 4 weeks stimulation, which may imply that vascular morphology change is sensitive to the duration of dynamic fluid stimulation. However, this result could not be overly interpreted based on the small number of samples. Overall, based on previous study on vessel ligation effects on bone adaptation [20], such small percentage of vessel wall changes in the nutrient vessel may not significantly affect the blood flow in bone. Nevertheless, further study will be needed to explore this mechanism.

SMCs are exposed to wall shear stress via the transmural pressure gradient [34,43]. It has been proposed that blood pressure affects transmural flow and able to regulate the normal cellular activities of SMCs, i.e., proliferation and migration [34,43-46]. Future studies will focus the relationship between the changes in mechanical environment due to ImP oscillations and the cellular responses of SMC, such as the coupling process of proliferation and apoptosis. Oscillatory shear stress has been shown to increase smooth muscle cell proliferation via protein kinase B phosphorylation and activate various signal transduction pathways [34,43-46]. Hypertensive rats model have demonstrated that reductions of blood flow augmented vessel wall hypertrophy via mechanisms that enhance SMC proliferation in the media and the intima [47]. While others have shown SMCs in mesenteric resistance arteries can undergo cell death in both low flow and high flow conditions [26]. These studies indicated that proliferation and apoptosis of SMCs may be involved in the remodeling of the nutrient artery.

Other potential SMC mechanisms related to the morphological changes seen in vascular adaptation are the change in size and arrangement of existing SMCs [26] However, there is considerable controversy regarding SMC hypertrophy and hyperplasia (increase in cell number such as via cell proliferation) in medial thickening of hypertensive models. Some studies have concluded medial thickening is due to SMC hyperplasia based on the observations of increased DNA content and numbers of SMCs [48]. While other studies have assessed cellular hypertrophy via morphometric estimation of cell size in tissue sections and measurements of protein to cell ratios, concluded medial hypertrophy is due to enlargement of existing SMCs [49].

Lastly, arterial morphological changes may also be a result of the changes in connective tissues. In hypertensive rats, results have shown a higher content of elastin in the arterial wall and an increase in polar amino acids content in elastin, which suggested that the material properties of the artery is altered due to the continuous physical stress that placed on the vessel from high blood pressure and increase of peripheral resistance [28,29]. Likewise for collagen, both the quantitative and qualitative changes were determined. Many experiments have shown the stimulation of collagen synthesis and the increase of collagen content in the arterial wall in hypertension [28,29,48]. In order to fully understand the processes of vascular remodeling, the above mechanisms are important for future studies.

## Conclusions

The adaptive response in the nutrient arteries was investigated via our avian model which can induce oscillatory fluid flow in the absence of bone matrix deformation. Bone fluid flow induced by ImP is a critical mediator for bone remodeling, possibly through altering blood supply to bone and disrupting the nutrient transport process. Stress fractures were often observed in young populations who had experienced high intensity physical training, i.e., athletes and military recruits. These data suggest that repetitive cyclic loading may trigger arterial wall enlargement, which may potentially reduce the fluid supply to bone and further generate local ischemia. Three-weeks of ImP stimulation was sufficient to increase arterial wall area, lumen area, wall thickness, and SMC layer numbers. The mechanical signals generated from ImP may ultimately initiate a cascade of cellular responses via mechanotransduction, influencing cellular activities within the arterial wall. With the strong interactions between blood flow and bone remodeling, it is highly suggestive that bone fluid flow has a potential to contribute to stress injuries to bone via an ongoing repair process.

## Competing interests

The authors declare that they have no competing interests.

## Authors' contributions

YXQ was the principle investigator who designed the overall study and carried out the surgical procedure. HL assisted during surgical procedure, participated in the daily stimulation, performed tissue and statistical analyses, and drafted the manuscript. PB provided suggestions on vessel physiology and biology analyses. All authors read and approved the final manuscript.
